# Food sufficiency status and sleep outcomes in older adults: the National Health and Aging Trends Study (NHATS)

**DOI:** 10.1186/s12937-024-00918-4

**Published:** 2024-02-27

**Authors:** Ashley C. Flores, Christopher Sarpong, Nan Dou, Muzi Na

**Affiliations:** 1 Department of Nutritional Sciences, 108C Chandlee Laboratory, The Pennsylvania State University, University Park, University Park, PA 16802 USA; 2 Department of Biology, The Pennsylvania State University, University Park, PA USA

**Keywords:** Nutrition, Health, Aging, Sleep disparity, Nationally Representative Data, NHATS

## Abstract

**Background:**

Studies investigating the relationship between food insecurity and sleep among older populations are limited. This study aimed to cross-sectionally examine the associations between food sufficiency status and sleep outcomes in a nationally representative sample of older adults.

**Methods:**

Our study included 1,665 older adults (≥ 65 years), using data from the 2013 and 2014 National Health and Aging Trends Study (NHATS). Food insufficiency was determined via participants’ experience and utilization of food assistance programs (FAP). Sleep outcomes, including nighttime and total sleep hours, sleep latency, and sleep quality, were derived from self-reported data. Multivariable linear regression and logistic regression models were used to estimate the associations between food sufficiency status and sleep outcomes.

**Results:**

In 2013–2014, 86.1% of older adults were classified as food sufficient without FAP, 9.85% as food sufficient with FAP, and 4.08% as food insufficient. Adjusting for sociodemographic characteristics, food sufficient older adults with FAP reported more total sleep hours (𝛽 = 0.31, 95% CI: -0.02, 0.64) than those participants who are food sufficient without FAP. Further adjusting for health factors, food sufficient participants with FAP had more nighttime sleep hours and greater total sleep hours compared to those participants food sufficient without FAP. Compared to those deemed as food sufficient without FAP, food sufficient participants with FAP had lower odds of having longer sleep latency (OR = 0.50, 95% CI: 0.28, 0.89), after further adjusting for physical function performance.

**Conclusions:**

Among older adults, food sufficiency with FAP is associated with greater total sleep hours, greater nighttime sleep hours, and lower odds of longer sleep latency. Our findings may help inform nutrition food assistance programs targeting older populations.

## Background

Food insecurity is characterized by limited access to adequate food as a result of a lack of funds and other additional resources [1]. In 2020, an estimated 5.2 million persons aged 60 years and over were food insecure, and 2.0 million were estimated as very low food security as captured by the set of 18 questions in the Food Security Supplement, used by the United States Department of Agriculture (USDA) [2]. Certain characteristics among particular groups of older adults are more likely to experience food insecurity, for instance, lower income, racial inequality, living with a disability or having functional impairments, social isolation, and chronic health conditions [2–5]. The estimated number of older persons, 65 years and over was 55.7 million in 2020 and is projected to increase to 94.7 million older adults by 2060 [6]. As the aging population continues to grow, older persons remain vulnerable in facing food insecurity highlighting the importance of understanding the nature of food insecurity among populations of advanced age.

In comparison to food secure older adults, food insecurity in older adults has been linked to worse health outcomes including fair or poor general health, depression, having at least one Activities of Daily Living (ADL) limitation, diabetes, high blood pressure, congestive heart failure, having a heart attack, gum disease, and asthma [7]. Food insecurity is also associated with lifestyle-related behaviors including outcomes pertaining to various aspects of sleep health [8–10], although prior work including older adult populations has been few [11]. Alterations among varying aspects of sleep have additionally been implicated in chronic conditions and health outcomes for populations of advanced age, for example, obesity [12], diabetes [13, 14], depression [15], cognition [16], and all-cause mortality and cardiovascular mortality [17]. Despite the importance of food security and sleep respective to health in older age, there is a scarcity of studies investigating the relationship between food insecurity status and sleep outcomes among older persons. One recent cross-sectional study of 1,201 participants in Ghana (aged $\ge$50 years), examined the relationship between food insecurity and poor sleep quality, finding that moderate and older adults with severe food insecurity were at higher risk of having poor sleep quality than those food insecure older adults [11]. However, little is known about the social determinants, in particular food insecurity, in relation to sleep disparities among older adults, illustrating an existing research gap in the current literature.

In this study, we aimed to examine food insecurity defined as food sufficiency status with several various sleep-related outcomes in a population of older adults. We cross-sectionally investigated the associations between food sufficiency status and different aspects of sleep including sleep quality, nighttime sleep duration, sleep latency, and total sleep hours (nighttime plus nap) in a nationally representative sample of older adults, aged 65 years and older.

## Methods

### Study sample

Initiated in 2011, The National Health and Aging Trends Study (NHATS) is an ongoing, longitudinal survey study of a nationally representative sample of Medicare beneficiaries, 65 years of age and older residing in the United States drawn from the Medicare enrollment file. The NHATS serves as a resource to characterize later life functions among older adults and decrease disability, promote health and independent functioning, and improve the quality of life in aging older populations.

Data were collected through annual interviews, in which participants completed standardized questionnaires to assess their socioeconomic status, mental and physical well-being, living arrangements, home environment, activities of daily life, and sleep behavior in addition to performance-based assessments of physical and cognitive capacity.

In wave 3 (2013) and wave 4 (2014), NHATS collected data on sleep duration, nap frequency and duration, sleep latency, and sleep quality by administering a sleep-focused module to a random subset of participants. Data were combined from these two rounds as a cross-sectional sample, yielding a total of 2915 participants (wave 3: *n* = 1621; wave 4: *n* = 1294) with available data on at least one of the sleep outcomes. After excluding participants with missing data on food sufficiency (*n* = 21) and other covariates on sociodemographic and health status (*n* = 1229), a total of 1665 participants were included in our final analytic sample.

### Assessment of food sufficiency status

As the NHATS survey data does not include an established standardized measure of food security or the validated USDA food insecurity measures, we used a summary indicator conceptualized by Tucher et al. [18] to identify the food sufficiency status among the NHATS participants. In NHATS, participants were asked to report their food insufficiency experience in the last month due to financial, social, and functional limitations (5 items). These indicators included going without groceries or personal items, going without a hot meal, and going without eating due to the lack of ability and social support in addition to skipping meals due to the lack of food available or money to purchase food and the number of days meals were skipped.

A summed score of the five items (0–5 points) was calculated as described previously [18], in which participants were categorized as food sufficient (FS, total score = 0 points) or food insufficient (FI, total score = 1 or more points). We further defined food sufficient participants by their use of food assistance programs. NHATS participants were asked in the last year whether they received any help from food assistance programs (FAP) (e.g., food stamps also called the Supplemental Nutrition Assistance Program or SNAP, Meals-on-Wheels). If food sufficient participants indicated yes, then they were considered FS with FAP whereas if food sufficient participants indicated no, then these participants were considered FS without FAP. In sum, participants were categorized as FS without FAP, FS with FAP, and FI.

### Assessment of sleep outcomes

The 4 sleep outcomes assessed in our study were represented as nighttime sleep hours, total sleep hours, sleep latency, and sleep quality. Nighttime sleep duration measures were based on the participant responses in hours rounded to the nearest whole number. Nap hours were assessed based on 2-item nap-related questions. Participants were first asked about their frequency of napping during the day within the last month. A response of “0” to nap hours was generated among participants who reported “never” on napping frequency. For participants reporting having taken naps, the corresponding nap hours were recorded. Total sleep hours during each day were calculated by summing up the nighttime sleep and nap hours. Sleep latency was accessed by having participants recall the time they spent falling asleep in the last month. Participants were categorized by having shorter sleep latency (≤ 0.50 h) or longer sleep latency (> 0.50 h). Additionally, sleep quality scores were created through 3-item questions on the frequency of taking more than 30 min to fall asleep, having trouble falling back asleep on nights after being awake, and taking medications to sleep in the last month. Each affirmative response was dichotomously coded, producing a range of 0–3 points in sleep quality measurements (where 0 = better sleep quality; 3 = worst sleep quality). Sleep quality was restricted to the participants with available data on at least one of the sleep outcomes. Participants were categorized as having high sleep quality (0 to 1 point) or low sleep quality (2 to 3 points).

### Assessment of covariates

Sociodemographic information including age, sex, race/ethnicity, education levels, marital status, income levels, and household size were available. The race/ethnicity of participants was grouped as white, non-Hispanic, black, non-Hispanic, Hispanic, and other. Education levels were categorized by participants’ highest degrees: obtained less than a high school diploma, received a high school diploma, and earned greater than a high school diploma. Marital status was determined by being married or living with a partner, and not married, widowed, separated, or divorced. Income level was assessed from 1 (lowest) to 5 (highest). Household size was represented by the number of people in the household. Further information regarding health factors included depression, anxiety, body mass index (BMI), hypertension, diabetes, and heart attack or myocardial infarction. Depression was evaluated by 2 items from the Patient Health Questionnaire-2 (PHQ-2) and anxiety was evaluated by 2 items from the Generalized Anxiety Disorder-2 (GAD-2) respectively. To screen for depression participants were asked over the last month to identify how often they (1) had little interest or pleasure in doing things and (2) felt down, depressed, or hopeless. To screen for anxiety participants were asked over the last month to identify how often they (1) felt nervous, anxious, or on edge and (2) been unable to stop or control worrying. Responses were represented as overall scores based on participant responses including not at all, several days, more than half the days, and nearly every day. Using self-reported height and weight measures, nutritional status as determined by BMI was classified as underweight (BMI < 18.5), normal (BMI 18.5–24.9), overweight (BMI 25.0–29.9), and obese (BMI > 30.0). Hypertension, diabetes, and heart attack or myocardial infarction diagnostic status were reported by participants and dichotomously coded. The physical function of the participants was determined by overall performance-based test scores as quartiles. To capture physical capacity, NHATS included five performance-based assessments. These assessments involved balance, usual walking speed, rapid chair stands, handgrip strength, and peak airflow. The scores ranged from 0 (worst) to 4 (best). Further details regarding measuring physical capacity and the specific test items have been reported previously [19].

### Statistical analyses

The mean (SD) and proportions describing respondents’ sociodemographic characteristics and health conditions were calculated and compared by FI status. Between-group differences in continuous (household size, depression score, and anxiety score) and categorical (age, sex, race/ethnicity, education, marital status, total household income quintiles, nutritional status, self-reported hypertension, diabetes, heart attack, or myocardial infarction, and physical function test score quartiles) variables were tested by the Kruskal–Wallis test and Chi-Square test, respectively. Multivariable linear regression models were applied to test the associations between FI status and sleep outcomes including nighttime sleep hours and total sleep hours per day. Multivariable logistic regression models were applied to test the associations between FI status and sleep outcomes including sleep latency and sleep quality. Model 1 was adjusted for age, sex, race/ethnicity, education levels, marital status, income levels, and household size. In model 2, health-related conditions including depression score, anxiety score, BMI, hypertension, diabetes, and heart attack or myocardial infarction were further adjusted. Model 3 was further adjusted for physical function performance test score quartiles. Statistical analyses were performed using Stata/SE (Version 15.0, College Station, TX).

## Results

The characteristics of NHATS participants by food sufficiency status are presented in Table 1. In this cross-sectional study of 1665 participants, 54.41% of participants were women and 8.53% of participants were 65–69 years of age. Participants were mostly non-Hispanic white (73.87%) and had educational attainment at or greater than the high school level (78.74%). The prevalence of FS without FAP in this study was 86.07% (*n* = 1433), whereas 9.85% of participants were considered FS with FAP (*n* = 164), and 4.08% of participants were FI (*n* = 68). Compared to the FS participants with and without FAP, FI participants were more likely to be women, to not be married, widowed, separated, or divorced, to be obese, have depression, anxiety, and have diabetes status.

**Table 1:** Sample characteristics of NHATS participants by food insufficiency status^1,2^

	All	FS without FAP	FS with FAP	FI	P-values
**N**	1665	1433	164	68	
**%**		86.07	9.85	4.08	
**Age groups, year**, **%**					0.88
65–69	8.53	8.44	10.37	5.88	
70–74	24.68	24.98	21.34	26.47	
75–79	22.94	22.54	26.22	23.53	
80–84	22.22	22.26	22.56	20.59	
85–89	13.27	13.33	10.98	17.65	
> 90	8.35	8.44	8.54	5.88	
**Females, %**	54.41	52.13	62.20	81.82	< 0.0001
**Race/Ethnicity, %**					< 0.0001
White, non-Hispanic	73.87	77.18	47.56	65.65	
Black, non-Hispanic	17.30	15.56	32.32	17.65	
Hispanic	3.06	3.07	3.05	2.94	
Other	5.77	4.19	17.07	11.76	
**Education, %**					< 0.0001
< High school diploma	21.26	17.10	51.22	36.76	
High school diploma	31.11	30.84	32.93	32.35	
> High school diploma	47.63	52.06	15.85	30.88	
**Marital status, %**					< 0.0001
Married, living with a partner	49.01	53.31	25.61	14.71	
Not married, widowed, separated, divorced	50.99	46.69	74.39	85.29	
**Income quintiles, %**					< 0.0001
1 (the lowest)	19.70	13.05	67.07	45.59	
2	21.38	20.52	26.22	27.94	
3	18.20	20.03	4.27	13.24	
4	19.46	21.98	1.83	8.82	
5 (the highest)	21.26	24.42	0.61	4.41	
**Household size**, **number of persons**	1.96 (1.08)	1.95 (1.03)	2.17 (1.38)	1.72 (1.13)	0.005
**Nutritional status, %**					< 0.0001
Underweight	2.10	2.09	1.22	4.41	
Normal	32.49	33.29	27.44	27.94	
Overweight	35.50	36.85	30.49	19.12	
Obese	29.91	27.77	40.85	48.53	
**Depression**, **score**	0.96 (1.39)	0.85 (1.29)	1.52 (1.72)	1.87 (1.72)	0.0001
**Anxiety, score**	0.85 (1.28)	0.76 (1.19)	1.34 (1.59)	1.57 (1.61)	0.0001
**Hypertension, %**	73.33	71.67	83.54	83.82	0.001
**Diabetes, %**	28.17	26.17	39.02	44.12	< 0.0001
**Heart attack or myocardial infarction, %**	2.82	2.37	6.10	4.41	0.02
**Physical function test score quartiles (*N*** **= 1448), %**		(*N* = 1258)	(*N* = 135)	(*N* = 55)	< 0.0001
1 (the worst)	22.44	18.68	48.15	45.45	
2	25.28	24.80	25.19	36.36	
3	29.49	30.76	22.96	16.36	
4 (the best)	22.79	25.76	3.70	1.82	
**Nighttime sleep** **(*N*** **= 1614), hours**		(*N* = 1396)	(*N* = 152)	(*N* = 66)	0.72
	7.05 (1.55)	7.06 (1.51)	7.09 (1.78)	6.83 (1.83)	
**Total sleep** **(*N*** **= 1518), hours**		(*N* = 1372)	(*N* = 146)	(*N* = 63)	0.82
	7.75 (1.77)	7.73 (1.73)	7.85 (1.98)	7.67 (2.06)	
**Sleep latency** **(*N*** **= 1584), hour**		(*N* = 1369)	(*N* = 154)	(*N* = 61)	0.01
	0.50 (1.01)	0.48 (1.03)	0.54 (0.76)	0.68 (0.86)	
**Sleep quality** **(*N*** **= 1649), score**		(*N* = 1420)	(*N* = 161)	(*N* = 68)	0.008
	1.11 (1.02)	1.08 (1.02)	1.25 (1.02)	1.44 (1.15)	

FAP, Food Assistance Programs; FI, Food Insufficient; FS, Food Sufficient; NHATS, The National Health and Aging Trends Study

^1^ Values are mean (SD) for household size, depression, anxiety, and sleep outcomes and % for other categorical variables

^2^ Between-group differences in continuous and categorical variables were tested by Kruskal –Wallis, and Chi-square tests, respectively

In model 1, FS participants with FAP had 0.31 more total sleep hours than FS participants without FAP (𝛽 = 0.31; 95% CI: -0.02, 0.64) after adjusting for sociodemographic characteristics, the significance level was marginal though (p-value = 0.062). In the full model (model 2), FS participants with FAP had 0.29 greater nighttime sleep hours (𝛽 = 0.29; 95% CI: 0.005, 0.57) than FS participants without FAP after further adjusting for health-related covariates. A marginal association was also observed that FS participants with FAP had 0.33 more total sleep hours (𝛽 = 0.33; 95% CI: -0.003, 0.66) than FS participants without FAP (p-value = 0.052). Additionally, FS participants with FAP had 0.62 odds of having longer sleep latency (> 0.50 h) in comparison to those FS without FAP, although the association was marginal (p-value = 0.064). We did not observe significant associations between food sufficiency status and sleep quality.

Among participants with available data on physical function performance (*n* = 1448), we further adjusted for their physical function scores (Table 2). Compared to participants who were FS without FAP, those who were FS with FAP had 0.50 odds of having longer sleep latency (> 0.50 h) (OR = 0.50; 95% CI: 0.28, 0.89). FI participants were not significantly associated with other sleep outcomes.

**Table 2:** The association between food sufficiency status and sleep outcomes among older populations (65+) using wave 3 and 4 NHATS data^1^

		Nighttime sleep hours b (95% CI)	Total sleep hours b (95% CI)	Sleep latency OR^5^ (95% CI)	Sleep quality OR^6^ (95% CI)
Model 1^1^	N	1614	1581	1584	1649
	FS without FAP	Ref	Ref	Ref	Ref
	FS with FAP	0.23 (-0.05, 0.52)	*0.31* *(-0.02, 0.64)*	0.75 (0.47, 1.21)	1.10 (0.75, 1.60)
	FI	-0.05 (-0.43, 0.34)	0.14 (-0.30, 0.59)	1.37 (0.75, 2.50)	1.18 (0.71, 1.96)
Model 2^2^	N	1614	1581	1584	1649
	FS without FAP	Ref	Ref	Ref	Ref
	FS with FAP	**0.29** **(0.005, 0.57)**	*0.33* *(-0.003, 0.66)*	*0.62* *(0.38, 1.03)*	0.95 (0.64, 1.41)
	FI	0.02 (-0.36, 0.41)	0.10 (-0.35, 0.55)	1.08 (0.57, 2.03)	0.88 (0.51, 1.51)
Model 3^3^	N	1411	1387	1389	1438
	FS without FAP	Ref	Ref	Ref	Ref
	FS with FAP	0.25 (-0.05, 0.54)	*0.32* *(-0.02, 0.67)*	**0.50** **(0.28, 0.89)**	0.85 (0.54, 1.32)
	FI	0.05 (-0.35, 0.45)	0.20 (-0.27, 0.66)	1.05 (0.52, 2.09)	0.95 (0.52, 1.72)

FAP, Food Assistance Programs; FI, Food Insufficient; FS, Food Sufficient; NHATS, The National Health and Aging Trends Study. Bolded indicates significant association. Italicized indicates marginal association

^1^ Multiple linear regression was used to test the associations between food insufficiency status with nighttime sleep hours and total sleep hours respectively. Multiple logistic regression was used to examine the associations between food insufficiency status and whether the participants had longer sleep latency and low sleep quality respectively

^2^ Model 1 was adjusted for age, sex, race/ethnicity, education levels, marital status, income levels, and household size

^3^ Model 2 was further adjusted for depression score, anxiety score, BMI, hypertension, diabetes, and heart attack or myocardial infarction

^4^ Model 3 was further adjusted for physical function performance test score quartiles

^5^ Sleep latency was considered as shorter sleep latency (≤ 0.50 h) or longer sleep latency (> 0.50 h)

^6^ Sleep quality was considered as high sleep quality (0 to 1 point) or low sleep quality (2 to 3 points)

## Discussion

In this study, we cross-sectionally examined food sufficiency status with various sleep outcomes among a nationally representative sample of older adults aged 65 and older. Among FS individuals with FAP, participants had more total sleep hours, greater nighttime sleep hours, and lower odds of having longer sleep latency in comparison to those participants FS without FAP contingent upon the adjustment for covariates such as sociodemographic information, health factors, or physical function performance. FI participants were not significantly linked to any sleep outcomes in our current study. These findings indicate that food assistance programs could play a role in decreasing the potential negative effects on health behaviors that arise with food insufficiency in older people, although future intervention studies are warranted to verify the findings.

To the best of our knowledge, previous observational studies conducted in older populations examining the relationship between food sufficiency status and sleep outcomes are limited. However, one recent cross-sectional study by Gyasi et al. [11] among 1,201 older adults (aged 50 years and older), found that older adults with moderate and severe food insecurity were significantly associated with poor sleep quality and also had fewer sleep hours in comparison to those food secure. Another cross-sectional study of 121 Latino adults with type 2 diabetes (mean age of 61 years) demonstrated that household food insecurity was significantly associated with worse sleep quality [20]. However, additional literature evaluating this relationship of interest is more established in other age groups including adult populations. A cross-sectional study by Ding et al. [8] investigated the relationship between food security status and sleep moderated by sex and included U.S. adults who participated in the National Health and Nutrition Examination Survey (NHANES) 2005–2010, demonstrating that very low food secure women in comparison to fully food secure women reported shorter sleep duration and marginally as well as very low food secure men reported longer sleep latency in contrast to fully food secure men. Jordan et al. [9] examined the relationship between household food insecurity status and sleep outcomes including sleep duration and sleep quality among adults in Mexico and found when compared with food secure households, severe household food insecurity was significantly associated with increased odds of getting less than 7–8 h of sleep an poor sleep quality. Despite the few studies conducted specifically among older age groups on the association between food insecurity and sleep outcomes, our study adds to the limited literature within the context of a nationally representative sample of older adults linking FS with FAP to sleep outcomes such as total sleep hours, nighttime sleep hours, and sleep latency.

The underlying mechanisms of the relationship between food sufficiency status and sleep are not completely clear. Although, several mechanisms including psychosocial, biobehavioral, and physiological factors may help to explain these associations. Among older adult populations, several studies have linked food insecurity to psychological mental distress [21] and depression [22–25]. Likewise, stress [26] and anxious or depressive appearances [27] have been noted in sleep disturbances. In the study by Bermúdez-Millán et al. [20], household food insecurity was associated with worse sleep quality through the mechanism of psychological distress among Latino participants with type 2 diabetes. Comparatively, Liu et al. [28] conducted a cross-sectional study of 12 U.S. states and also illustrated food insecurity was associated with insufficient sleep even after adjustment for frequent mental distress. As mental health has been implicated as an underlying mechanism between food insecurity and sleep and may impact these associations of interest in our study, we adjusted for depression and anxiety in our analyses and were still able to observe that FS participants with FAP in comparison to those FS without FAP, had greater nighttime sleep hours and more total sleep hours. Interestingly, a recent longitudinal study using NHATS data from 2012 to 2020, found that food insecurity and SNAP participation were associated with the rate of cognitive decline among older adults [29]. Given these findings, it may also be possible that cognitive deficit is mediated by a change in sleep behaviors as sleep and cognitive function appear to be related in older age [30, 31].

Furthermore, another potential mechanism impacting the relationship between food sufficiency status and sleep outcomes may stem from a nutritional component. Studies have shown that older adults who are food insecure have overall poorer diet quality [32, 33], lower intake of key nutrients [34], and increased risk of malnutrition [35]. The study by Lee and Frongillo, Jr [4] demonstrated that food insecure U.S. elderly persons had lower dietary intake as well as poorer nutritional and health status in contrast to food secure older persons. Likewise, inadequate intakes of a few key micronutrients including calcium, magnesium, and vitamins A, C, D, and E were related to sleep concerns [36]. Diet and sleep have also been implicated in a bidirectional relationship through hormonal pathways, energy intake, circadian rhythms, and nutrients and food intake [37]. Although diet quality may mediate the relationship between food insecurity and sleep health, NHATS does not contain a dietary indicator assessment. The absence of dietary data restricts our understanding of the role of diet quality on sleep for older adults participating in FAP versus those not participating in FAP. Therefore, we are limited by the available data to further explore this potential relationship.

Additionally, among older populations, prior studies have indicated a connection between food insecurity and functional limitations [38–40]. This association suggests that older adults who are food insecure may experience problems with limited food affordability, lack of food availability, and inaccessibility to the acquisition of food in addition to alterations in food use [3]. Subjective poor sleep is associated with physical disability among older adults [41]. To address the role functional limitations may play in food insecurity and sleep among elderly persons, we further adjusted for participants’ physical function based on in-person performance-based assessments. After adjustment for physical function performance, we found that FS participants with FAP, in contrast to those FS without FAP, had lower odds of having longer sleep latency. As our results changed when further adjusting for physical function performance in our analyses, some of this variance may potentially be explained by the influence of functional limitations on both food sufficiency status and sleep health. The underlying mechanisms between food insecurity and various sleep outcomes concerning older adults should continue to be explored to gain a better understanding of the nature of this relationship.

Our cross-sectional study has several strengths. Our study included a population of older adults from wave 3 and wave 4 of NHATS, a large nationally representative sample of older adults enrolled in Medicare, and followed annually through in-person interviews. Additionally, our sample included comprehensive information on sociodemographic characteristics, health-related factors, and physical function performance-based information. These covariates were adjusted in our analyses to control for potential confounding factors that may impact the observed associations shown. Further, we used a summary indicator to capture the food sufficiency status of participants that incorporated three domains of functional, social, and financial constraints for food acquisition as conceptualized specifically for the NHATS population [18]. In addition, we were able to further classify FS individuals by their use of food assistance programs which provides additional insight into the potential benefits of these programs on sleep health among older adults. Lastly, we were able to investigate a range of specific sleep outcomes highlighting the link between food sufficiency status and different aspects of sleep.

However, there are several limitations to address in our study. First, our study relied on self-reported data from participants to assess food sufficiency status and the various sleep outcomes, therefore, the data may be subject to recall bias. Although we used a summary indicator of food sufficiency status established in a nationally representative sample of older adults, we acknowledge the current gold standard for measuring food insecurity is the USDA Household Food Security Survey Module (HFSSM). However, the summary indicator conceptualized by Tucher et al. [18] was able to expand upon the concept of food sufficiency status specific to older adults using the NHATS population within the context of financial limitations, functional impairments, and social constraints. In addition, the various sleep outcomes assessed in our study were based on subjective measures of participants’ self-reported responses. The use of subjective measures of sleep such as questionnaires based on self-reported responses in comparison to objective measures of sleep (e.g., polysomnography, accelerometry) may demonstrate varied findings among older adults [42, 43]. Due to limitations in the NHATS data, we were unable to adjust for environmental features (e.g., noise, light, temperature, etc.) [44, 45] which may influence sleep. Similarly, we were unable to adjust for the influence of adequate nutrition as a confounding factor despite recognizing the interconnectedness between diet and sleep [46]. Our findings when comparing FS with FAP participants in contrast to those FS without FAP participants should be interpreted cautiously, as the characteristics of participants who enroll versus those who do not enroll in FAP may not be fully controlled for. As our study utilized a cross-sectional design, we were unable to examine the temporal relationship between food sufficiency status and sleep outcomes. Therefore, the direction of these associations cannot be identified. Additional longitudinal studies and intervention studies are warranted to better characterize the direction of food sufficiency status and sleep health.

## Conclusions

In conclusion, our findings suggest that better sleep outcomes (greater total sleep and nighttime sleep hours, and longer sleep latency) were associated with FS individuals who reported having food assistance in the fully adjusted model. Factors such as socioeconomics, health status, health behavior, and physical function were necessary adjustments to determine a difference comparing FS adults with and without FAP. Our findings may provide additional support for participation in food assistance programs among aging populations, which has been historically low [47, 48]. Future studies conducted in older age groups with larger sample sizes, longitudinal study designs, diverse populations, validated food insecurity measures, and the use of subjective and objective measures of sleep outcomes are warranted.

## Data Availability

The datasets generated and analyzed during the current study in the manuscript are publicly and freely available without restriction in the National Health and Aging Trends Study repository (https://www.nhats.org/researcher/data-access). The code book and analytic code will be made available upon request pending approval by the corresponding coauthor.

## Abbreviations

BMI: body mass index
FAP: food assistance programs
FI: food insufficient
FS: food sufficient
NHATS: National Health and Aging Trends Study
USDA: United States Department of Agriculture
